# InDel marker detection by integration of multiple softwares using machine learning techniques

**DOI:** 10.1186/s12859-016-1312-2

**Published:** 2016-11-02

**Authors:** Jianqiu Yang, Xinyi Shi, Lun Hu, Daipeng Luo, Jing Peng, Shengwu Xiong, Fanjing Kong, Baohui Liu, Xiaohui Yuan

**Affiliations:** 1 School of Computer Science and Technology, Wuhan University of Technology, Wuhan, China; 2 School of Computer Science and Technology, Heilongjiang University, Harbin, China; 3 The Key Lab of Soybean Molecular Design Breeding, Northeast Institute of Geography and Agroecology, Chinese Academy of Sciences, Harbin, China

**Keywords:** Insertions and deletions, InDel detection, Evaluation

## Abstract

**Background:**

In the biological experiments of soybean species, molecular markers are widely used to verify the soybean genome or construct its genetic map. Among a variety of molecular markers, insertions and deletions (InDels) are preferred with the advantages of wide distribution and high density at the whole-genome level. Hence, the problem of detecting InDels based on next-generation sequencing data is of great importance for the design of InDel markers. To tackle it, this paper integrated machine learning techniques with existing software and developed two algorithms for InDel detection, one is the best F-score method (BF-M) and the other is the Support Vector Machine (SVM) method (SVM-M), which is based on the classical SVM model.

**Results:**

The experimental results show that the performance of BF-M was promising as indicated by the high precision and recall scores, whereas SVM-M yielded the best performance in terms of recall and F-score. Moreover, based on the InDel markers detected by SVM-M from soybeans that were collected from 56 different regions, highly polymorphic loci were selected to construct an InDel marker database for soybean.

**Conclusions:**

Compared to existing software tools, the two algorithms proposed in this work produced substantially higher precision and recall scores, and remained stable in various types of genomic regions. Moreover, based on SVM-M, we have constructed a database for soybean InDel markers and published it for academic research.

## Background

Molecular markers play a key role in population genetics and evolutionary studies, as well as in the construction of genetic maps [1]. The development of molecular markers has undergone various stages, including restriction fragment length polymorphism (RFLP), single-strand conformation polymorphism (SSCP), random amplified polymorphism detection (RAPD), amplified fragment length polymorphism (AFLP), short simple tandem repeats (SSR) [2], single nucleotide polymorphisms (SNPs) [3, 4], and short insertions and deletions (InDels) [1]. Among them, InDels are widely distributed in genomes [1, 5]. When compared with the other types of markers, InDels are characterized by their lengths that are generally less than 50 bp [6], whereas the other types of markers are with lengths larger than 50 bp. Therefore, InDel markers can be easily detected and it is for this reason that they are commonly used as genetic markers [1, 7].

In order to construct a complete database of InDel markers, there is a necessity to develop approaches that are capable of detecting InDel markers accurately. In recent years, a number of software products for InDel detection have been devleoped, such as Samtools [8], GATK UnifiedGenotyper (GATK-UG) [9], Pindel [10], SOAPIndel [11], VarScan [12], SplazerS [13], and Dindel [14]. The main difference among these software tools lies in the models they use to identify InDel markers. In particular, Samtools and GATK-UG investigate the results of alignment between sequencing data and the reference genome, and employ different Bayesian statistical models to calculate the posterior probability of the genotype at each locus for InDel detection. Pindel uses unmapped reads in the alignment results and applies a pattern growth algorithm to detect InDel variations. Varscan is based on the pileup data from Samtools, and uses a heuristic algorithm to detect InDel variations, and it can also handle problems such as extreme read depth, as well as pooled and contaminated samples. SOAPIndel uses a De Bruijn graph algorithm to recombine all unmapped reads, and detects InDel variations according to the alignment with the reference genome.

However, there is no such standard method for InDel detection that can ensure a promising performance in terms of accuracy, and each of popular detection softwares has its own advantages and disadvantages in terms of their performances of precision and recall. Furthermore, certain simple strategies are generally adopted by software tools to improve the performance of the detection results. Taking Samtools as an example, the values of read depth are utilized as a quality control to filter out inaccurate results, as higher read depths usually indicate problematic regions which are often enriched for incorrect InDel markers [15]. Moreover, these strategies often increase the rate of false negative InDel markers. Nevertheless, as has been pointed out by [16], the performances of the software tools mentioned above are not satisfactory as indicated by their low scores in the measures of precision and recall.

Hence, to improve the performances of existing software tools, a number of computational approaches that integrate with these software tools have been developed to provide more accurate detection results. For example, HugeSeq Pipeline integrates with GATK-UG and Samtools for the purpose of detecting SNP/InDels, but when detecting Structural Variation/Copy Number Variation (SV/CNV), HugeSeq Pipeline prefers to utilize Pindel, CNVnator [17], Breakdancer [18], and BreakSeq [19]. It combines the results so as to improve the performance in terms of recall, and extracts common detection results to improve the precision performance [20]. Based on SNP detection results obtained from multiple software tools, BAYSIC uses a Bayesian algorithm to improve the accuracy of the results [21]. However, BAYSIC cannot be used for the detection of InDels. HugeSeq only integrates with two software tools, namely Samtools and GATK-UG, and the optimization strategy is relatively simple so that it can only detect relatively small InDels (1–8 bp).

In addition to the integration with existing softwares, machine learning techniques have also been recently applied in variation detection. SVM2 [22] and SV-M [23] are developed based on SVM. ForestSV [24] follows the random forest algorithm. Platypus [25] integrates the results from multiple software tools and optimizes the screening by using a genetic algorithm. However, the recall performance of using SVM2 for detecting heterozygous variations is not satisfactory as indicated by low scores [18]; SV-M is insufficient for detecting insertions, as it is only capable of detecting insertions within a length range of 2–5 bp; forestSV can only detect relatively large insertions (>50 bp) and CNVs, but not InDels; and Platypus can only detect SNPs, but not InDels.

To the best of our knowledge, none of existing software tools that integrate with machine learning techniques has been proposed specifically for InDel detection. However, motivated by the promising performance of such strategy when used to detect other variations (SNPs, SVs, and CNV), we have reason to believe that the strategy of integrating machine learning techniques with existing software tools can also be able to improve the accuracy of InDel detection.

To detect InDel markers in a more accurate and comprehensive manner by using the strategy mentioned above, we propose two InDel detection methods: BF-M algorithm, which is based on the optimal F-score that considers both precision and recall to measure the accuracy, and SVM-M algorithm, which is designed according to SVM. Both BF-M and SVM-M are developed as a general tool for the detection of InDel markers and can be applied to the genomes of all species. The experimental results show that with BF-M, detection results with high F-score can be obtained, and the detection results of SVM-M are characterized by the highest recall and F-score. Finally, we used SVM-M to detect InDels in soybeans collected from 56 different regions, and screened these by selecting highly polymorphic loci to construct a soybean InDel marker database.

## Methods

### Programs for the simulation of variation and sequencing

**Fig. 1:**
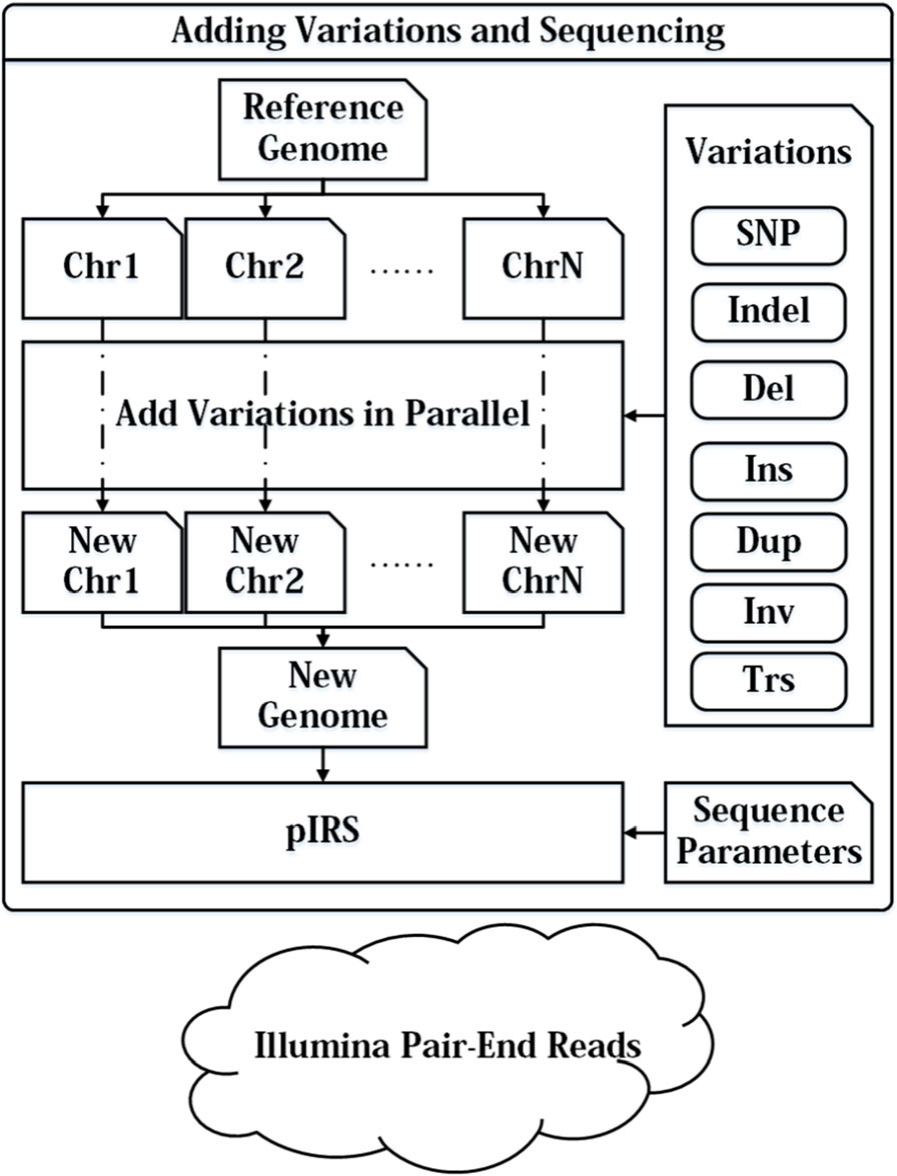
Flowchart of variation and sequencing simulations

To demonstrate the performances of existing software tools from the perspectives of precision and recall, specific information on the variations should be acquired, such as location, size and characteristics of genome sequence segments where variations are located. Moreover, based on such information, it is also possible for us to evaluate the influences made by the characteristics of the genome sequence on the detection results. Hence, we used computer simulation to add known variations to the reference genome so as to generate new genomic sequences, and then used this sequencing simulation technology to generate the sequence data as described in Fig. 1. The program for variation simulation was developed by our group with C++ language, and the program pIRS [26] was used for sequencing simulation.

### Generation of the training set and the test set (simulation data)

The data of the training set and the test set were both generated by using the following parameters. The reference genome used in the generation process was soybean Williams82 (Gmax_189), and its InDels were composed of SNP, large fragments of insertions/deletions, duplications, inversions, and translocations as presented in Table 1. The parameters of sequencing are listed in Table 2.

**Table 1:** Variation distribution

Variation Type	Size(bp)	Number
SNP	1	1/1000
Indel	1–50	2792000
Deletion/Insertion	51–500	20000
Duplication	100–500	1000
Inversion	100–500	1000
Translocation	100–500	1000

**Table 2:** Sequencing parameters

Sequencing Depth	Read Length	Insert Size	Standard Deviation
5X	100	500	100

### Sequence alignment and InDel detection

Using BWA [27], the sequencing data and the reference soybean genome (William 82) were aligned to generate the sam files, and samtools view was employed to convert the sam files into bam files. The bam files were sorted by coordinates with the tool of samtools sort, repeats were removed by using samtools rmdup, and then indexed by using samtools index. Next, the five software tools were utilized for variation detection. For Varscan, the parameter "minimum sequencing depth" was set to 2; for the remaining four software tools, parameter defaults were employed. Finally, InDels within a length range of 1–50 bp were extracted.

### Software selection

For approaches based on software integration, if the mutual verification and complementation among the results obtained from these software tools are more related, the screening results will be better. In this work, we chose to use Samtools, GATK-UG, Varscan, Pindel, and SOAPIndel to generate the original InDel data. Among these five software tools, Samtools, GATK-UG and Varscan make use of mapped regions to detect InDel markers, whereas the other software tools utilize the un-mapped regions to do so. In addition, through simulation studies, we found that the detection results from these five software tools can provide complementary verification with each other.

### Criterion for determining consistent results

Through simulation experiments, we found that the results of different softwares showed the existence of deviation between coordinates of InDels when they were used to detect the same InDel markers. Such deviation can be ascribed to the similarity between sequences. For example, when detecting an AT deletion from the sequence ATATAT, the software may report a deletion of any of the three AT dinucleotides. However, since the proposed algorithms identify InDel markers by merging the identification results obtained from multiple software tools, it is possible for our algorithms to identify all the three regions as InDel markers. In particular, we use (1) to calculate the coordinate deviation between the results obtained from different softwares. It should be noted that the coordinate of an InDel marker is the starting position where the InDel is found in a genome sequence. The statistical analysis [1, 15] indicates that in the soybean genome, the range of coordinate deviation is in non-repeat regions, and is less than or equal to the length of the repeat sequence in repeat regions. Therefore, we set the criterion of result consistency as variation sequences with same size, which in turn forces the coordinate deviation falling within the above ranges. Finally, the difference in the coordinates of two detected InDel markers can be computed using the following equation:

1 $$ D=\left|P1-P2\right| $$

where *P1* is the coordinate of an InDel, and *P2* is the coordinate of the other InDel. A smaller value of *D* denotes that the two InDel markers are more close to each other in the genome sequence.

### The details of BF-M

The BF-M algorithm is composed of three steps:

1) The common part in the detection results obtained from all pairs of software tools is grouped according to the attributes of InDels; 2) for each group F-score is calculated; and 3) the group with the best F-score is selected as the optimization rule.

#### Selection of the grouping attributes

InDels have four important attributes, including variation type (ST), variation size (SS), the type of the repeated region where the variation is located (RT), and detection software (DS). The detection results are then grouped according to these four attributes. In particular, G(F,S) is hereby used to denote a set of groups resulted from grouping S in terms of the attribute F. Each group corresponds to a specific value of F. Therefore, G(ST,S) denotes a set of groups obtained by grouping a set of InDel markers, denoted by S, in terms of the attribute ST. For the case of multiple grouping operations, if S is first grouped in terms of the attribute ST and then grouped again based on the attribute SS, we can use G(SS,G(ST,S)) to represent the groups resulted from such grouping procedure. Each group in G(SS,G(ST,S)) corresponds to a specific combination of attribute values of SS and ST.

**Fig. 2:**
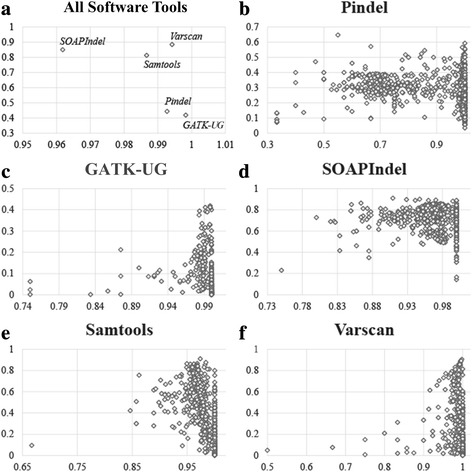
Distribution of precision and recall scores of five different software tools

For InDel with the same type of repeat sequence and the same size, the data of simulation experiments indicated that in G(DS,G(RT,G(SS,G(ST, detection results)))), the detection result obtained from each software performed differently in terms of precision and recall. In Fig. 2, the horizontal axis represents the precision score, and the vertical axis indicates the recall score. Fig. 2a shows the distribution of precision and recall scores using the five software tools for detecting a non-repetitive 1-bp deletion. In Fig. 2a, GATK-UG yielded the best precision score (99.83 %) but with the smallest recall score (41.92 %), whereas Varscan obtained the highest recall score (88.42 %). This finding suggests that the selection of software is an important factor to the accuracy of detection. In addition, for the same software, the precision and recall scores when detecting InDels of different types of repeat sequence and different sizes also vary significantly according to Fig. 2b-f, which describe the distribution of precision and recall scores of the five software tools in the different groups of G(SS,(ST, detection results)). To quantitatively demonstrate the difference in the performance of recall and precision, we computed the standard deviation of recall and precision for each of software tools when applying them to detect InDel markers from different groups of G(SS, G(ST, detection results)). In particular, based on the results we used to draw Fig. 2b-2f, the standard deviations of precision for the software tools Pindel, GATK-UG, SOAPIndel, Samtools and Varscan are 0.15, 0.03, 0.01, 0.01 and 0.01 respectively, while the standard deviations of recall are 0.07, 0.1, 0.03, 0.25 and 0.26 for the software tools Pindel, GATK-UG, SOAPIndel, Samtools and Varscan respectively. The large standard deviations in precision and recall indicate that the dispersion in the performances of all groups is very large in both precision and recall. Hence, a conclusion can be reached that the selection of attributes plays a crucial rule on the performance of detecting InDels.

#### The optimization rule

**Fig. 3:**
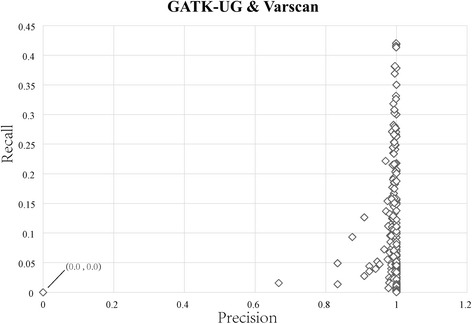
Distribution of precision and recall scores of common results detected by GATK-UG and Varscan

Generally speaking, the common part in the detection results of multiple software tools is believed to be able to improve the performance in terms of precision. However, it can be observed from Fig. 3 that the efforts made by the common part to the precision score are not always positive. In particular, assuming that IR denotes the common part in the detection results of GATK-UG and Varscan, we first obtained a set of groups by following G(SS, G(ST, IR)) and then applied each of groups to detect InDel markers. The performance of each group is presented in Fig. 3 using the symbol in the shape of diamond. In this regard, the coordinate of a diamond symbol describes the precision and recall scores for the corresponding group. From Fig. 3, we find that some groups show relatively high precision and recall scores as their corresponding diamond symbols are located in the top right corner of Fig. 3, whereas some other groups show high precision scores but their recall scores are rather low. It is also noted that there is one group whose diamond symbol locates in the bottom left corner, which means that the precision and recall scores of this group are very close to 0. Hence, directly combining all IRs will still include groups with unsatisfactory performance in terms of precision and recall, thus preventing achieving the best performance.

Observing the distribution of precision and recall scores of common InDel markers detected by GATK-UG and Varscan in Fig. 3, the worst performance was found in the detection of 21-bp deletions within SSR regions, in which case both precision and recall scores were 0.

**Fig. 4:**
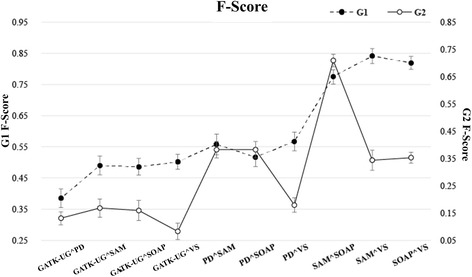
The performance in terms of F-score for the detection results of 1-bp deletions and 9-bp deletions

After analyzing all IRs, we found that for InDels detected by the same ST and SS, the precision and recall scores of detection results did not change much across different software tools. In Fig. 4, G1 shows the F-scores of the detection results on 1-bp deletions within TIR regions using different IRs, G2 shows the F-scores of the detection results for 9-bp deletions within SSR regions using different IRs. The best F-scores of Samtools and Varscan on G1 were much close, and the best F-scores of Samtools and SOAPInDel on G2 were also similar. For a 1-bp deletion of low complexity, the common results shared by GATK-UG and Pindel obtained the highest precision score, but its recall scores were the worst. Similarly, the common part in the detection results of Samtools and SOAPIndel showed the lowest precision scores but their recall scores were the best. Therefore, selecting the result with the highest precision score will lead to unsatisfactory performance in terms of recall, and vice versa.

### The details of SVM-M

SVM maps eigenvectors to a high-dimensional space by using a kernel function, and splits the data in this space to construct two parallel hyperplanes. The distance between the two parallel hyperplanes is maximized to build an optimized classification model, and this model is used for data classification. We chose the libsvm software package [28] developed by Chih-Jen Lin as the SVM classifier.

#### Selection of the eigenvector

We constructed an eigenvector that contained five eigenvalues including the software used for InDel detection, InDel type, InDel length, the type of repeat sequence where the InDel is located, and the number of reads that match InDel detection results.

When an InDel is only detected by one software tool, the number of reads is the value that this software generates. On the other hand, when an InDel is detected by multiple software tools, the number of reads is the sum of the values that all these software tools produce.

The SVM type we chose is C-SVC (support vector clustering) in libsvm, and for kernel function, we chose the radial basis function (RBF) defined by (2). By using this method, the values of two parameters, C and γ, were determined to obtain the optimal classification results. However, for a given problem, no priori experiences can be applied to determine the values of C and γ. Based on the training dataset, we used the Cross-validation and Grid-search functions provided by libsvm to search the parameter space to determine the optimal values of C and γ, and then used these parameter values and the training set to generate the final classifier. In the candidate training set, the number of InDels was huge, and it was time-consuming if all these InDels were used as the training data. Therefore, we employed the subset.py script provided by libsvm to select 100,000 InDels as the training set to train the SVM classifier. We also used OpenMP to modify the code for libsvm in order to support parallel functions, thereby effectively improving the computational efficiency.

2 $$ \mathrm{R}\mathrm{B}\mathrm{F}= \exp \left(-\gamma \ast \left|u-v\right|2\right) $$

**Fig. 5:**
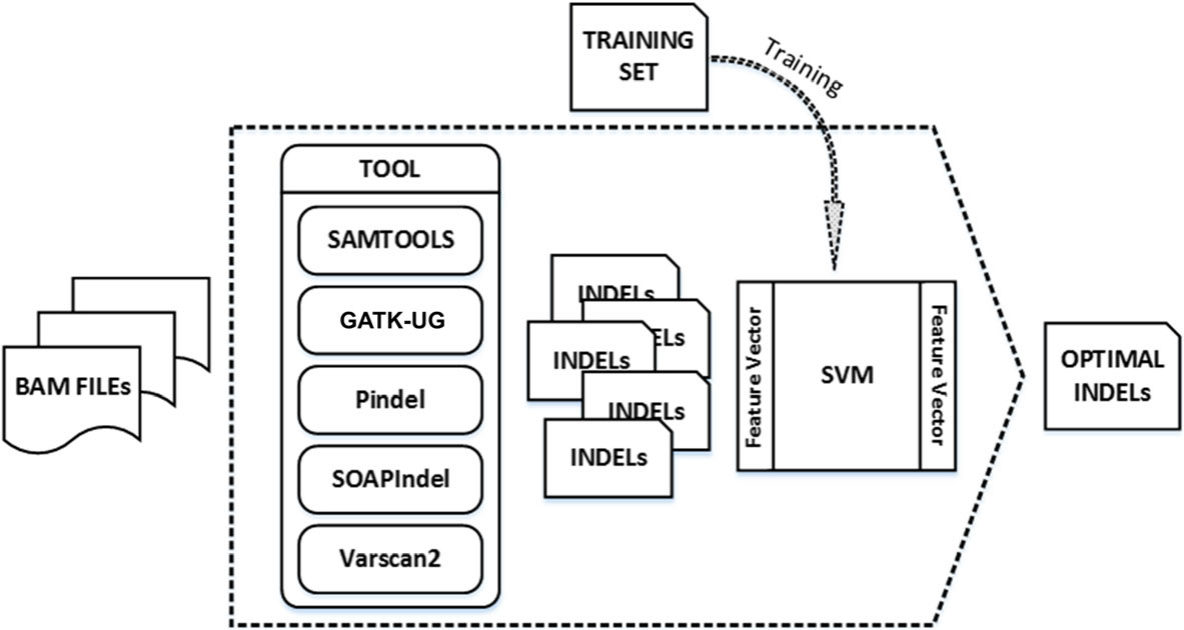
Optimal InDel screening method based on SVM

In (2), *u* and *v* are the eigenvectors we construct for InDel markers. The flowchart for the SVM-based InDel optimization screening method is shown in Fig. 5.

## Results and discussion

### Experiment setup

To evaluate the performances of BF-M and SVM-M, we compared them against five software tools, including Samtools, GATK-UG, PIndel, SOAPindel and Varscan. Regarding the parameter setting for these five softwares, we adopted the default setting as provided by the corresponding authors of softwares. Although we admitted that the parameters were of significance to determine the performance of software. However, it is time-consuming for users to tune the values of parameters in order to achieve the best performance. In this regard, default parameters were used as they were recommended by the authors of the software to ensure a satisfactory performance when using the software. In addition, another reason why we selected default parameters for software tools is to demonstrate the robustness of the proposed algorithms. Since the proposed algorithms integrate the results of multiple software tools, the influences made by the parameters of individual software tools are trivial to the performance of the proposed algorithms. In this regard, no matter what parameters we select for the software tools involved, our algorithms can still obtain a promising performance as indicated by the experimental results.

**Fig. 6:**
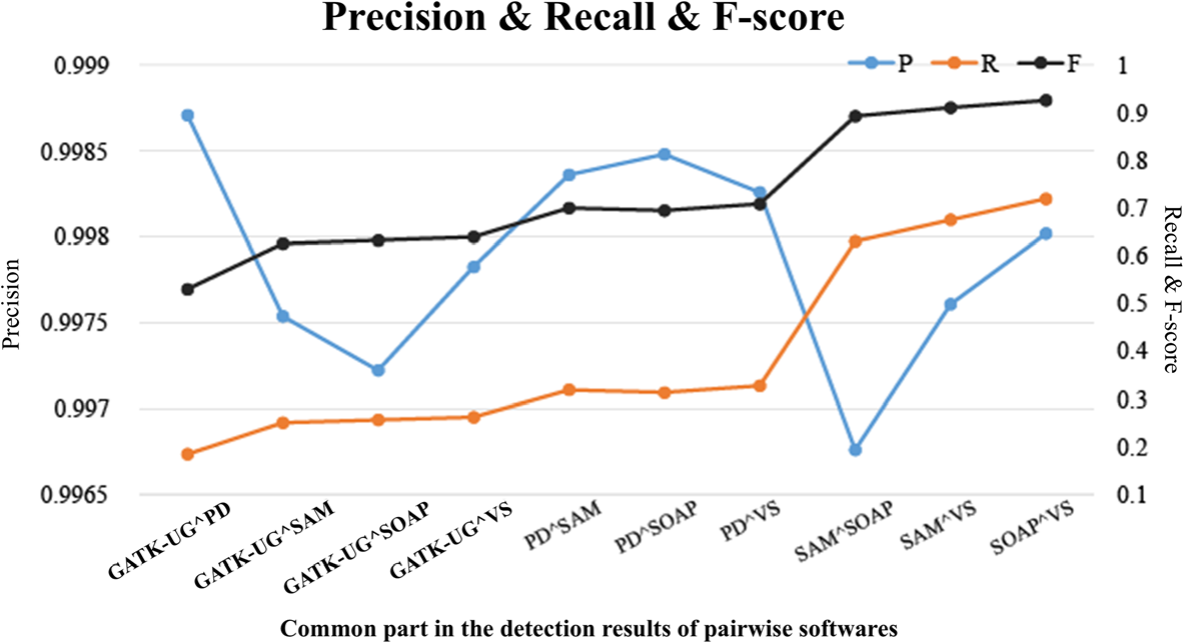
The performance of precision, recall, and F-score for the common part in the detection results of pariwise softwares

The F-score, defined by (3), is an important indicator for assessing the balance between precision and recall. Simulation experiments show that the F-scores of G(RT, G(SS, G(ST, IR))) exhibited a stable pattern of change, and the best F-score was found in a different IR for all the groups as described in Fig. 6. Based on these findings, we selected the combination of attributes with the best F-score among groups of the same RT, SS, and ST values in G(RT,G(SS,G(ST,IR))) as the optimization rule, and used this to screen the detection results.

3 $$ \mathrm{F}-\mathrm{score}=2\times \frac{\mathrm{precision}\times \mathrm{recall}}{\mathrm{precision}+\mathrm{recall}} $$

In (3), precision is the number of correct positive results divided by the number of all positive results, and recall is the number of correct positive results divided by the number of positive results that should have been returned. When used to measure the accuracy of InDel detection, a high precision score means that a detection algorithm returns substantially more correct InDel markers than incorrect, while high recall means that a detection algorithm returns most of correct InDel markers.

### Evaluation of the performances of BF-M and SVM-M

In this work, we used simulation data to assess the two proposed methods. We added 2,792,000 InDels with length 1–50 bp to the reference of soybean genome (Gmax_189), and simulated the Illumina paired-end sequencing data. InDels with length 1–50 bp detected by the five selected software tools were then subjected to the screening using BF-M and SVM-M. The precision scores, the recall scores, and the F-scores obtained by the different methods were then compared as indicated in Table 3.

**Table 3:** The performance of precision, recall, and F-score for each software tool

Indels						
Tool	Precision(%)	Diff *	Recall (%)	Diff	F-score (%)	Diff
BF-M	99.32	0.00	65.20	0.00	78.72	0.00
SVM-M	95.75	−3.57	84.56	19.37	89.81	11.09
GATK	99.49	0.17	25.50	−39.70	40.59	−38.13
Pindel	94.69	−4.63	41.36	−23.84	57.57	−21.15
Samtools	97.46	−1.86	65.71	0.51	78.49	−0.23
SOAPIndel	97.25	−2.07	74.74	9.54	84.52	5.80
Varscan	98.59	−0.73	64.66	−0.54	78.10	−0.62
Deletions						
Tool	Precision(%)	Diff	Recall (%)	Diff	F-score (%)	Diff
BF-M	99.21	0.00	66.34	0.00	79.51	0.00
SVM-M	95.85	−3.37	84.84	18.50	90.01	10.50
GATK	99.40	0.19	26.03	−40.30	41.26	−38.25
Pindel	89.89	−9.32	39.06	−27.28	54.45	−25.06
Samtools	97.78	−1.43	66.32	−0.02	79.03	−0.48
SOAPIndel	96.86	−2.35	75.43	9.09	84.81	5.30
Varscan	98.55	−0.66	65.17	−1.17	78.46	−1.05
Insertions						
Tool	Precision(%)	Diff	Recall (%)	Diff	F-score (%)	Diff
SVM-M	95.66	−3.77	84.29	20.23	89.62	11.69
GATK	99.59	0.16	24.96	−39.10	39.92	−38.00
Pindel	99.45	0.01	43.66	−20.40	60.68	−17.24
Samtools	97.14	−2.29	65.09	1.03	77.95	0.03
SOAPIndel	97.64	−1.79	74.05	9.99	84.23	6.31
Varscan	98.64	−0.80	64.15	0.09	77.74	−0.18

*Diff denotes the difference between each software tool and BF-M in terms of Precision, Recall and F-score

For all InDels, the precision score obtained by BF-M was higher than those obtained by Samtools, Pindel, SOAPIndel, and Varscan. On the other hand, the recall score was higher than GATK-UG and Pindel. With SVM-M, the precision score was higher than that obtained by Pindel, and the recall score and F-score were higher than those obtained by all five software tools.

For deletions, the precision score of BF-M was higher than Pindel, Samtools, SOAPindel, and Varscan, and its performance in terms of recall was much better than GATK-UG and Pindel. On the other hand, the precision score of SVM-M was higher than that of Pindel, and its recall score and F-score were higher than those of the five software tools.

For insertions, the precision performance of BF-M was better than Samtools, SOAPindel and Varscan, and its recall score was much higher than those of GATK-UG and Pindel. The performance of SVM-M in terms of recall and F-score was better than all five software tools.

Regarding the length of the detected variations, GATK-UG only detected deletions 1–37 bp in length and insertions 1–25 bp in length; Samtools could only detect deletions 1–44 bp in length and insertions 1–29 bp in length; and Varscan exclusively detected deletions 1–42 bp in length and insertions 1–28 bp in length. In contrast, both BF-M and SVM-M were capable of detecting InDels of 1–50 bp in length.

**Fig. 7:**
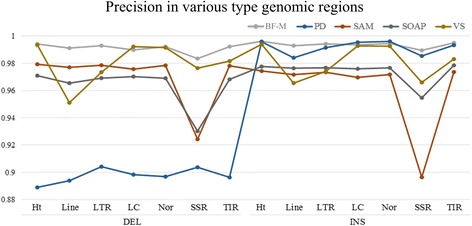
Precision of different types of genomic regions

**Fig. 8:**
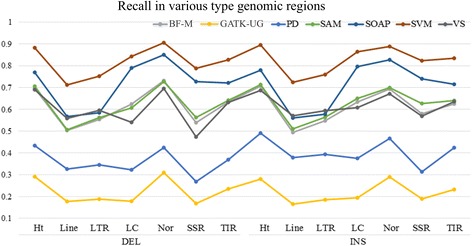
Recall of different types of genomic regions(Ht: Helitron, LC: Low complexity, SSR: Simple repeat, Nor: Non-repeat region, F1: BF-M method, PD: Pindel, SAM: Samtools, SOAP: SOAPIndel, SVM: SVM-based method, VS: Varscan)

**Fig. 9:**
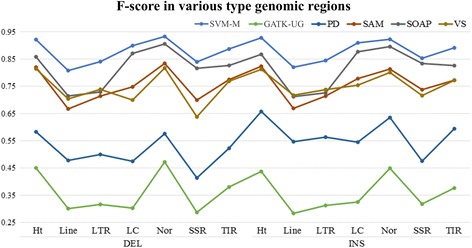
F-score on different types of genomic regions(Ht: Helitron, LC: Low complexity, SSR: Simple repeat, Nor: non-repeat region, SVM: SVM-M, PD: Pindel, SAM: Samtools, SOAP: SOAPIndel, VS: Varscan)

Repeat sequences are composed of several short repeats that can have a significant impact on the precision of variation detection. Therefore, we assessed the InDel detection results of repeat regions obtained by using various methods. For all the five software tools, both precision and recall scores declined on repeat sequences when compared with those of non-repeat regions. In particular, the long interspersed nuclear elements (LINEs), long terminal repeat (LTRs), and simple repeats showed the most substantial decreases in precision scores for most of the software tools as described in Fig. 7. For deletions, compared with non-repeat regions, the precision scores of LINEs, LTRs, and simple repeats as generated by Varscan were reduced by 4.08 %, 1.82 %, and 1.52 %, respectively; with Samtools and SOAPindel, the precision scores for simple repeats were reduced by 5.53 % and 4.01 %, respectively. For insertions, compared to non-repeat regions, the precision scores of LINEs, LTRs, and simple repeats by using Varscan were reduced by 2.73 %, 1.87 %, and 2.68 %, respectively; with Samtools and SOAPindel, the precision scores for simple repeats decreased by 7.76 % and 2.25 %, respectively. In contrast, the precision scores obtained with BF-M were apparently stable for all sequence types. The smallest decrease in precision score was observed in simple repeat regions, and compared to non-repeat regions, it was only reduced by 0.88 %. In addition, although the precision scores of BF-M were higher than those of Pindel, Samtools, SOAPindel, and Varscan for LINEs, LTRs, simple repeats, and terminal inverted repeats (TIRs), the performance of SVM-M in terms of recall and F-score was better than all the five independent software tools as indicated by Figs. 8 and 9.

### Design and application of soybean InDel markers

Of the 56 soybean varieties collected from different regions around the world, 27 were originated from Northeast China, eight were from a study conducted by Li et al. [29], 15 were from an investigation led by Chung et al. [30], and six first employed by Kim et al. [31].

By using SVM-M, a total of 742,977 InDels with a length range of 5–50 bp were detected. Among them, 21,452 highly polymorphic InDel loci were selected and annotated by using the software Annovar. The results of the annotation are presented in Table 4.

**Table 4:** Statistical analysis of InDel markers

Position	Number
intergenic	12266
promoter	2379
downstream	1852
UTR3	540
UTR5	571
exonic	517
intronic	3309
splicing	18
total	21452

By using the automated batch primer design function as provided in the software Primer3 [32], we designed the upstream and downstream primers for 21,452 InDel loci. The specificity of the primers to genomic sequences was considered in the analysis to improve the success rate of the designed molecular markers.

## Conclusions

In this work, software integration and machine learning algorithms were utilized in designing the BF-M and SVM-M algorithms. The precision and recall scores of BF-M reached 99.32 % and 65.19 %, respectively; the precision and recall scores of SVM-M were 95.75 % and 84.56 %, respectively, and the F-score was 89.81 %. For deletions, the precision and recall scores of BF-M were 99.21 % and 66.34 %, respectively; the precision and recall scores of SVM-M were 95.85 % and 84.84 %, respectively. For insertions, the precision and recall scores of BF-M were 99.43 % and 97.14 %, respectively, and the precision and recall scores of SVM-M were 95.66 % and 84.29 %, respectively. In addition, for InDel detection within repeat sequences, BF-M showed a high precision score and stable performance, whereas SVM-M maintained the highest recall and F-score when compared with the other software tools. These results suggest that compared against individual software tools, the two algorithms proposed in this study produced substantially higher precision and recall scores, and still remained stable in various types of genomic regions. Finally, based on SVM-M, we have constructed a database for soybean InDel markers.

The optimized algorithms proposed in this study have no special requirements for the type and number of InDel detection software tools. Additional software can be added to this InDel detection technology to further improve the performance of the proposed algorithms.

## Abbreviations

AFLP: Amplified fragment length polymorphism
BF-M: Best F-score method
CNV: Copy number variation
DP: Read depth
DS: Detection software
InDel: Insertions and deletions
LINE: Long interspersed nuclear element
LTR: Long terminal repeat
RAPD: Random amplified polymorphism detection
RBF: Radial basis function
RFLP: Restriction fragment length polymorphism
RT: Type of the repeat region where the variation is located
SNP: Single nucleotide polymorphisms
SS: Variation size
SSCP: Single-strand conformation polymorphism
SSR: Short simple tandem repeats
ST: Variation type
SV: Structural variation
SVM: Support vector machine
SVM-M: Support vector machine method
TIR: Terminal inverted repeats
